# ILC2 Lung-Homing in Cystic Fibrosis Patients: Functional Involvement of CCR6 and Impact on Respiratory Failure

**DOI:** 10.3389/fimmu.2020.00691

**Published:** 2020-05-07

**Authors:** Anja Schulz-Kuhnt, Vicky Greif, Kai Hildner, Lisa Knipfer, Michael Döbrönti, Sabine Zirlik, Florian Fuchs, Raja Atreya, Sebastian Zundler, Rocío López-Posadas, Clemens Neufert, Andreas Ramming, Alexander Kiefer, Anika Grüneboom, Erwin Strasser, Stefan Wirtz, Markus F. Neurath, Imke Atreya

**Affiliations:** ^1^Department of Medicine 1, University Hospital of Erlangen, Erlangen, Germany; ^2^Department of Medicine 3, University Hospital of Erlangen, Erlangen, Germany; ^3^Department of Pediatrics and Adolescent Medicine, University Hospital of Erlangen, Erlangen, Germany; ^4^Department of Transfusion Medicine and Haemostaseology, University Hospital of Erlangen, Erlangen, Germany

**Keywords:** group-2 innate lymphoid cells, cystic fibrosis, CCR6, type-VI collagen, tissue remodeling

## Abstract

Cystic fibrosis patients suffer from a progressive, often fatal lung disease, which is based on a complex interplay between chronic infections, locally accumulating immune cells and pulmonary tissue remodeling. Although group-2 innate lymphoid cells (ILC2s) act as crucial initiators of lung inflammation, our understanding of their involvement in the pathogenesis of cystic fibrosis remains incomplete. Here we report a marked decrease of circulating CCR6^+^ ILC2s in the blood of cystic fibrosis patients, which significantly correlated with high disease severity and advanced pulmonary failure, strongly implicating increased ILC2 homing from the peripheral blood to the chronically inflamed lung tissue in cystic fibrosis patients. On a functional level, the CCR6 ligand CCL20 was identified as potent promoter of lung-directed ILC2 migration upon inflammatory conditions *in vitro* and *in vivo* using a new humanized mouse model with light-sheet fluorescence microscopic visualization of lung-accumulated human ILC2s. In the lung, blood-derived human ILC2s were able to augment local eosinophil and neutrophil accumulation and induced a marked upregulation of pulmonary type-VI collagen expression. Studies in primary human lung fibroblasts additionally revealed ILC2-derived IL-4 and IL-13 as important mediators of this type-VI collagen-inducing effect. Taken together, the here acquired results suggest that pathologically increased CCL20 levels in cystic fibrosis airways induce CCR6-mediated lung homing of circulating human ILC2s. Subsequent ILC2 activation then triggers local production of type-VI collagen and might thereby drive extracellular matrix remodeling potentially influencing pulmonary tissue destruction in cystic fibrosis patients. Thus, modulating the lung homing capacity of circulating ILC2s and their local effector functions opens new therapeutic avenues for cystic fibrosis treatment.

## Introduction

Cystic fibrosis (CF) represents the most common inherited disease in Caucasians and an epidemiological study performed in 2016 even predicts an increase of about 50% in the overall number of patients diagnosed for this life-threatening disease by the year 2025 in Western European countries (1, 2). The pathophysiology of CF is based on defined mutations in the cystic fibrosis transmembrane conductance regulator (*CFTR*) gene, which cause dysfunction of the CFTR chloride channel and, subsequently, an impaired epithelial chloride and bicarbonate transport resulting in viscid mucus production (3, 4). Although CF pathology involves various organ systems, respiratory failure as consequence of progressive lung disease appears as the leading cause of disease-associated morbidity and mortality (3–5).

Fibrosis and bronchiectasis represent key characteristics of pulmonary CF manifestation (4, 6, 7). It is well-accepted that the etiology of bronchiectasis depends on a complex interplay between chronic lung infections, progressive pulmonary tissue remodeling and an accumulation of pro-inflammatory immune cells (8). Mainly as consequence of viscid mucus production and impaired mucociliary clearance, airways of CF patients are highly susceptible to bacterial colonization and infection. This permanent crosstalk of microbial pathogens with the CFTR-dysfunctional airway epithelium triggers a pro-inflammatory but yet inefficient mucosal immune response (4, 9) dominated by a marked accumulation of neutrophils (10). Due to a complex dysregulation of adaptive and innate immune cell function and interplay (11, 12), the lung tissue, bronchoalveolar lavage (BAL) and sputum of CF patients are characterized by increased levels of inflammation-promoting chemokines and cytokines, including for instance IL-8, GM-CSF, IL-6, CCL2, CCL3, CCL4, CCL20, TNF-α, IL-1β, IL-17, IL-23, G-CSF, IL-9, and IL-33 (10, 13–16). Within this inflammatory milieu, neutrophil-derived elastase and matrix metalloproteinases represent crucial mediators of extracellular matrix degradation and CF-associated pulmonary tissue remodeling (17–19). In addition to chronic airway infections as inflammatory triggers, immune responses have been suggested to be partly driven by intrinsic alterations independent from non-self recognition in CF patients (20) and thus gained new research interest.

During the last decade, innate lymphoid cells (ILCs) emerged as rare but exceptionally potent representatives of the innate immune system. They are of particular importance for host defense at epithelial barrier surfaces (21), but also contribute to the systemic pool of circulating immune cells (22, 23). Characterized by a type-2 cytokine profile and the expression of signature surface molecules (e.g., CD127 and CRTH2) (24, 25) and transcription factors (e.g., GATA3) (26), group-2 ILCs (ILC2s) represent a predominant helper ILC population in the lung under steady state conditions (27, 28). There they are preferentially located in direct proximity to the airway epithelium or accumulate within infected foci (27, 29). This predisposes pulmonary ILC2s for sensing epithelial injury and participating in a first line of immunological defense against invading pathogens. In case of allergic or infectious lung diseases, inflammatory mediators like IL-33, IL-25, and thymic stromal lymphopoetin are locally released by damaged epithelial cells and potently stimulate ILC2 activity (30–35). Mainly via the secretion of characteristic cytokines (e.g., IL-5, IL-13, IL-9, and IL-4) and growth factors, but also based on cell contact-dependent mechanisms, activated ILC2s promote type-2 immune responses, support mucosal wound healing and, thereby, crucially impact on the maintenance and reconstitution of tissue homeostasis (27, 36–40). Overwhelming ILC2 activation, however, was found to be involved in chronic inflammation, allergy, and fibrotic tissue remodeling (24, 41, 42). Although published RNA-seq. data from bronchial brush samples implicated that the lung pathology in CF is predominated by Th17 and Th1 gene signatures (43), the increased pulmonary presence of the ILC2-activating cytokine IL-33 and augmented levels of the type-2 effector cytokines IL-5, IL-9, and IL-13 in CF BAL and sputum samples as well as the upregulation of local Th2 responses upon chronic infections with *Pseudomonas aeruginosa* in CF patients strongly argued for a potential, albeit less elucidated, involvement of ILC2s in CF pathogenesis (13, 14, 42, 44–46). Accordingly, the risk of asthma, a prototypical ILC2-initiated allergic disease (47), was found to be significantly higher in CF patients compared to non-carriers of a *CFTR* mutation (48), implicating exaggerated ILC2 activities in CF. In line with this, *Cftr*^−/−^ mice showed elongated ILC2 responses compared to C57Bl/6 wild type mice upon infection with *Aspergillus fumigatus* (14). Furthermore, mainly based on analyses in preclinical murine models with CF-like pathology, Moretti et al. demonstrated that ILC2-derived IL-9 triggers an auto-amplifying pro-inflammatory cycle via activation of mast cells, which in turn supports ILC2 functions by producing the growth factor IL-2, indicating an orchestrating role of lung-resident ILC2s in CF-associated inflammation (14). However, the direct clinical relevance of ILC2 function for pulmonary manifestation of human CF disease as well as the origin of activated lung ILC2s remain undefined. Therefore, we here analyzed the functional significance of circulating human ILC2s in the peripheral blood (pb) for the development of CF–associated fibro-inflammatory changes in the lung. To address this, we examined pb ILC2 function in CF by taking advantage of human blood samples and *in vivo* studies in a new humanized mouse model for ILC2 lung homing. Our results identified the CCR6 - CCL20 axis as regulator of pulmonary ILC2 migration and suggest local ILC2 activation as a potential driver of pulmonary type-VI collagen production in CF patients.

## Materials and Methods

### Human Blood Samples

After informed written consent, peripheral blood was collected in EDTA-coated tubes from patients with cystic fibrosis (*n* = 59), inflammatory bowel diseases (*n* = 19), and rheumatoid arthritis (*n* = 17), as well as healthy control subjects (*n* = 61). Characteristics of all study subjects are summarized in Table S1. Patient material was obtained from the Department of Medicine 1 and 3 as well as the Department of Pediatrics and Adolescent Medicine of the University Hospital of Erlangen, Germany. Leukocyte cones were derived from the Department of Transfusion Medicine and Haemostaseology of the University Hospital of Erlangen, Germany. Blood donation was approved by the local ethical committee and the institutional review board of the University of Erlangen-Nuremberg, Germany.

### Primary Human Blood Cell Isolation

Peripheral blood mononuclear cells (PBMCs) were isolated from whole blood, leukocyte cones and buffy coat blood via density gradient centrifugation using Pancoll human (PAN-Biotech) or Lymphocyte separation media (Anprotec). Where indicated, PBMCs were further enriched for CD4^+^ or CRTH2^+^ cells using magnetic bead-based isolation according to the manufacturer's instructions (Miltenyi Biotec).

### Flow Cytometric Characterization of Human ILCs

To identify human ILC2s, ILC1s, and ILC3s, single cell suspensions were treated with FcR blocking reagent (Miltenyi Biotec) before incubation with the following fluorochrome-conjugated anti-human antibodies: hematopoietic lineage cocktail [eFlour450, including CD2 (RPA-2), CD3 (OKT3), CD14 (61D3), CD16 (CB16), CD19 (HIB19), CD56 (CB56), and CD235a (HIR2), eBioscience], CD11c (VioBlue, MJ4-27G12, Miltenyi Biotec), CD127 (APC-Vio770, REA614, Miltenyi Biotec), CD161 (FITC, 191B8, Miltenyi Biotec), CD7 (FITC, CD7-6B7, BioLegend), CD117 (APC, 104D2, BioLegend), and CRTH2 (PE, BM16, Miltenyi Biotec). To further analyze human ILC subgroups, specific antibodies targeting CCR4 (APC, L291H4, BioLegend), CCR5 (Alexa Flour 647, HEK/1/85a, BioLegend), CCR6 (PE/Cy7, G034E3, BioLegend), CCR9 (PerCP/Cy5.5, L053E8, BioLegend), CXCR3 (APC, G025H7, BioLegend), CD4 (PerCP/Cy5.5, OKT4, BioLegend), CD45 (APC, HI30, BioLegend), CD69 (APC, FN50, BioLegend), CD123 (PerCP/Cy5.5, 6H6, BioLegend), TCRα/β (APC, IP26, BioLegend), TCRγ/δ (APC, B1, BioLegend), and respective isotype control antibodies were used. Surface-stained cells were uniformly fixed in 1x BD CellFix (eBioscience) according to the manufacturer's specifications or measured directly after staining. For intracellular staining, the Foxp3/Transcription Factor Staining Buffer Set (eBioscience) in combination with a specific fluorochrome-conjugated antibody targeting human GATA3 (APC, REA174, Miltenyi Biotec) was used. LSR Fortessa (BD Bioscience) or MACSQuant 10 (Miltenyi Biotec) cell analyzers allowed data acquisition. For further data processing, the FlowJo single cell analysis software 7.6.5 and 10.06.1 (Tree Star Inc.) was used. Samples with <20 acquired ILC2s were excluded from further ILC2 phenotyping.

### *Ex vivo* Expansion and Stimulation of Human pb ILC2s

To *ex vivo* expand human pb ILC2s, they were isolated from total PBMCs, derived from CF patients or healthy volunteers, via fluorescence-activated cell sorting (FACS) as Lin^neg^CD127^+^CD161^+^CRTH2^+^ lymphoid cells using a FACSAria II (BD Bioscience) or a MoFlo Astrios (Beckman Coulter) cell sorter. PBMCs from 3 different donors were γ-irradiated (45 Gray) and pooled to serve as feeder cells. Sort-purified human ILC2s (100/well) were then co-cultured with feeder cells (4 × 10^5^/well) in 96 well-round bottom plates in 200 μl Yssel's Medium with 1% human serum AB (BioConnect) and 1% penicillin/streptomycin (P/S) per well. ILC2 expansion was stimulated with phytohaemagglutinin (PHA, 1 μg/ml, Sigma), rh IL-2 (100 IU/ml, Miltenyi Biotec), rh IL-25 (50 ng/ml, eBioscience) and rh IL-33 (50 ng/ml, BioLegend). Medium and cytokines were replenished every 3 to 4 days for 13–15 days. Afterwards, the ILC2 expansion factor was calculated based on flow cytometric enumeration of Lin^neg^ CD127^+^CD161^+^CRTH2^+^ ILC2s per well. Expanded ILC2s were then rested in rh IL-2 (20 IU/ml, Miltenyi Biotec) alone for 2 days. For restimulation, expanded and rested cells were re-seeded at 1 × 10^5^ or 3 × 10^5^ cells/well and stimulated with rh IL-2 (100 IU/ml, Miltenyi Biotec), rh IL-25 (50 ng/ml, eBioscience) and rh IL-33 (50 ng/ml, BioLegend) for 3 additional days or overnight, respectively. Supernatants collected between day 11 and 15 of the expansion phase or after 3 days of restimulating adjusted cell numbers of expanded control subject-derived and CF-derived ILC2s were used as ILC2 conditioned media in fibroblast experiments as indicated. Conditioned medium of stimulated feeder cells alone served as control.

### Enzyme Linked Immunosorbent Assay (ELISA)

Cytokine concentrations in cell culture supernatants derived from restimulated human ILC2s were quantified via ELISA analyses of human IL-4 (BioLegend), IL-5 (eBioscience, Invitrogen), IL-9 (BioLegend) and IL-13 (eBioscience, Invitrogen). ILC2 conditioned supernatants were used undiluted or diluted up to 1:1000. The optical density was measured using a NOVOstar plate reader (BMG Labtech).

### *In vitro* Transmigration Assay

To analyze the migratory capacity of circulating human ILC2s under defined experimental conditions, freshly isolated CRTH2^+^ human blood cells were labeled with the hematopoietic lineage cocktail (eFlour450, eBioscience) and fluorescent antibodies targeting CD11c (VioBlue, MJ4-27G12, Miltenyi Biotec), CD161 (FITC, 191B8, Miltenyi Biotec) and CRTH2 (PE, BM16, Miltenyi Biotec). 1.6 × 10^5^ stained CRTH2^+^ cells were suspended in 80 μl *X-Vivo* 15 medium (Lonza, 1% P/S) and applied to the upper insert of a 96 well-plate with 3 μm pore size (Corning). If indicated, a CCR6 blocking antibody (50 μg/ml in the insert, MAB195, R&D) or the respective isotype control antibody (BioLegend) were added. The bottom well was filled with 235 μl *X-Vivo* 15 medium and rh CCL20 (10 ng/ml and 100 ng/ml, Immunotools) as indicated. CCL25 (100 ng/ml, Immunotools) served as negative control, PGD2 (10 nM, Merck) as positive control (31). After 4 h at 37°C the number of migrated ILC2s in the bottom chamber was determined as Lin^neg^CD161^+^CRTH2^+^ lymphoid cells via flow cytometry. The migration index was defined as relative migration of Lin^neg^CD161^+^CRTH2^+^ cells attracted toward a chemotactic stimulus compared to those attracted by medium alone.

### Animals and Papain-Induced Airway Inflammation

C57BL/6 mice were housed under specific pathogen-free conditions in individually ventilated cages with a regular day-night cycle. For the induction of lung inflammation, age-matched mice were anesthetized by *i.p*. injection of ketamine/xylazin and were intranasally treated with 50 μg papain (Merck) for 3 consecutive days. All experiments involving animals were approved by the Government of Lower Franconia, Germany.

### *In vivo* Lung Homing Assay

To investigate the migratory capacity of human blood cells under *in vivo* inflammatory conditions, CD4^+^ human cells or expanded, rested and overnight restimulated human ILC2s with a purity of at least 95% of Lin^neg^ cells were used. If necessary, magnetic bead-based enrichment of Lin^neg^ cells was performed (Miltenyi Biotec). Prior to injection, cells were labeled with the cell proliferation dye eFlour670 (eBioscience) and resuspended in 1x phosphate buffered saline (PBS). 1.5 × 10^5^ to 1 × 10^6^ labeled cells were injected intravenously into the tail vein of C57BL/6 mice with papain-induced lung inflammation. Mice without cell transfer served as negative control. If indicated, 2 μg of rh CCL20 (Immunotools) was administered intranasally to anesthetized mice 15 min prior to cell transfer. After 24 h of recirculation, mice were euthanized and the pulmonary circulation was perfused with 5 mM EDTA in 1x PBS via the right ventricle. For the collection of BAL, lungs were carefully flushed three times with PBS supplemented with 0.1 mM EDTA via the trachea. Obtained BAL cells were labeled with fluorochrome-conjugated antibodies targeting CD11c (VioBlue, N418, Miltenyi Biotec) and SiglecF (PE-Vio770, ES22-10D8, Miltenyi Biotec) in order to flow cytometrically determine BAL eosinophils. To inflate the lung tissue to its physiological size prior to removal, 0.75% agarose or 4% paraformaldehyde (PFA) was inserted via the trachea as demonstrated earlier (49). The lung tissue and ileum, if indicated, were then harvested and further subjected to the indicated analyses. During lung perfusion, peripheral blood was collected and erythrocytes were lysed using ammonium-chloride-potassium lysis buffer (155 mM ammonium chloride; 19 mM potassium hydrogen carbonate and 0.68 mM EDTA; pH 7.27) allowing the flow cytometric identification and characterization of injected labeled cells retained in the blood circulation. In order to differentiate between live and dead cells, blood cells were stained with the fixable viability dye eFlour405 (eBioscience) and fixed with the Foxp3/Transcription Factor Staining Buffer Set (eBioscience) prior to flow cytometry.

### Light-Sheet Fluorescence Microscopy

For light-sheet microscopic visualization of lung-accumulated human blood ILC2s, the lung tissue was prepared as described elsewhere (50). In short, lung lobes were fixed in 4% PFA for 2 h at 4°C under constant rotation. Dehydration was performed in an ascending ethanol series of 50, 70, and 100% (2x) for at least 4 h each. Dehydrated samples were then cleared with ethyl cinnamate (Sigma) at room temperature and imaged with the UltraMicroscope II (LaVision, BioTec). 3D reconstruction and quantification of accumulated human blood ILC2s within the pulmonary tissue were performed with the Imaris Image Analysis software 9.0.2 (Bitplane) as described in detail earlier (49). Accumulated labeled cells were counted in 2 to 3 lung cubes with defined volume per lung with a mean cell count of 156 labeled ILC2s per lung cube.

### Immunohistochemistry

Cryosections of murine lung tissue were fixed in 4% PFA followed by blocking with 5% BSA, Roti-ImmunoBlock (Carl Roth) and donkey serum. Primary antibodies targeting Col VI (1:250, EPR17072, Abcam), Col I (1:200, polyclonal, Abcam) or myeloperoxidase (MPO, 1:100, Abcam) were incubated overnight. Collagens were then stained with the secondary antibody donkey anti-rabbit Cy3 (1:200, Poly4064, BioLegend) that was incubated for 1 h at room temperature. Slides treated with the secondary antibody alone served as control. For MPO staining, slides were incubated with a donkey anti-rabbit biotin antibody (1:200, Dianova), followed by Streptavidin-Cy3 (1:200, BioLegend). Nuclei were counterstained using Hoechst 33342 (ThermoFisher Scientific). Image acquisition was performed with the fluorescence microscope DM600B (Leica) for quantitative images and the confocal microscope SP8 (Leica) for representative images. Data analysis and quantification were performed with Fiji (National Institutes of Health). Therefore, the signal intensity of peribronchial Col VI and Col I relative to the Hoechst signal was determined. MPO^+^ cells were counted manually.

### Fibroblast Culture

Primary human lung fibroblasts, fetal (Sigma) were cultured in DMEM/F12 medium (Gibco) supplemented with 10% heat-inactivated fetal bovine serum (FBS), 1% P/S, 0.5% L-glutamine and 0.2% amphotericin B and were maintained at 37°C in a humidified incubator containing 5% CO_2_. Prior to stimulation, 1 × 10^5^ fibroblasts were seeded per well of 6 well-plates and rested in 0.1% FBS overnight. Fibroblasts were then stimulated for 48 h with fresh medium supplemented with rh IL-4 (1 pg/ml and 5 pg/ml, Immunotools) and rh IL-13 (10 ng/ml, Immunotools) or 1:5 diluted conditioned ILC2 supernatants derived from *ex vivo* stimulated human ILC2s (collected between d11 to d15 or after 3 days of restimulating adjusted numbers of expanded ILC2s). When indicated, anti-human IL-4 (10 ng/ml, eBioscience), anti-human IL-13 (20 ng/ml, BioLegend) or combinations of these were added. Conditioned supernatant derived from stimulated feeder cells alone or 1:5 diluted Yssel's medium (1% human serum AB, 1% P/S) supplemented with the cytokine cocktails used for ILC2 expansion (PHA, IL-2, IL-33 and IL-25) or restimulation (IL-2, IL-33, and IL-25) served as control. Fibroblasts up to passage eleven were used.

### Immunoblotting

Proteins were isolated from tissue or cells by using mammalian protein extraction reagent (ThermoFisher Scientific) containing protease and phosphatase inhibitors (Roche Diagnostics). Protein concentrations were determined via Bradford assay (Carl Roth) or using the Nanodrop 2000 spectrophotometer (ThermoFisher Scientific). Proteins were then denatured at 99°C in NuPAGEsample Buffer (Life Technologies) and separated by SDS-PAGE gels. After blotting proteins onto nitrocellulose membranes, blocking was performed in 5% non-fat milk. Membranes were incubated overnight at 4°C with the following primary antibodies: Col VI (Abcam), Col I (Cell Signaling Technology), phospho-STAT3 (Cell Signaling Technology), STAT3 (Cell Signaling Technology), phospho-STAT6 (Cell Signaling Technology) and STAT6 (Cell Signaling Technology). β-Actin (Cell Signaling Technology) and tubulin (Cell Signaling Technology) served as loading controls. If necessary, a horseradish-peroxidase conjugated secondary antibody (goat anti-rabbit IgG, Cell Signaling Technology) was incubated for 1 h at room temperature. Chemiluminescence was detected using the ECL Western blotting substrate (ThermoFisher Scientific) by the Amersham Imager 600 (GE Healthcare Bio-Sciences). Quantification of protein levels relative to the loading control was performed using Fiji (National Institutes of Health).

### Statistical Analysis

Statistical analyses were performed using the Prism 8 software (GraphPad). Either the two-tailed Student's *t*-test with Welch correction, if necessary, or the Mann-Whitney test was performed. Relative data were analyzed using the one-sample *t*-test. If indicated, single outliers were detected by the Grubbs' test (α = 0.05). Results are displayed as bar graphs or scatter plots indicating mean ± SEM. Correlations were calculated by the Spearman or Pearson test as indicated. *P* < 0.05 were considered statistical significant in all tests with asterisks indicating the following levels of significance: ^*^p < 0.05, ^**^p < 0.01, ^***^p < 0.001, ^****^p < 0.0001.

## Results

### Advanced Respiratory Failure Is Associated With a Decrease of Circulating CCR6^+^ ILC2s in CF Patients

**Figure 1:**
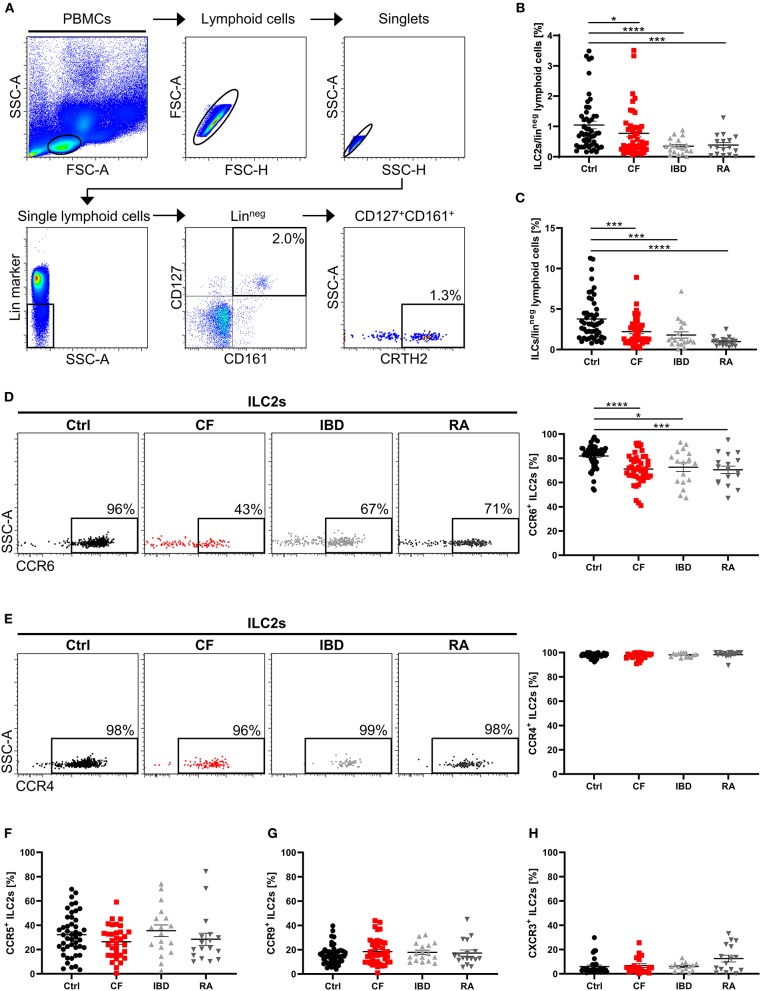
Altered ILC2 profile in the peripheral blood of patients with chronic inflammatory diseases. **(A)** Representative gating scheme for the flow cytometric identification of human pb ILC2s as Lin^neg^CD127^+^CD161^+^CRTH2^+^ lymphoid cells in total PBMCs. Percent values indicate frequencies of gated cell populations on Lin^neg^ lymphoid cells. **(B)** Quantified proportion of pb ILC2s among Lin^neg^ lymphoid cells in the peripheral blood from healthy controls (ctrl) and patients suffering from cystic fibrosis (CF), inflammatory bowel diseases (IBD) and rheumatoid arthritis (RA) (*n* = 17–50). **(C)** Quantified percentage of total ILCs (Lin^neg^CD127^+^ lymphoid cells) on Lin^neg^ lymphoid cells (*n* = 17–49). **(D,E)** CCR6 (*n* = 17–49) and CCR4 (*n* = 14–39) expression on human pb ILC2s in ctrl, CF, IBD and RA subjects; representative gating and corresponding quantification. **(F–H)** Quantification of the CCR5 (*n* = 17–46), CCR9 (*n* = 17–46) and CXCR3 (*n* = 14–31) expression on pb ILC2s. Statistical significance was determined using the Mann–Whitney test. Single outliers across all cohorts were detected via the Grubbs' test. *p < 0.05, ***p < 0.001, ****p < 0.0001.

Recent insights into the *in vivo* behavior of ILC2s implicate that these cells are present in the systemic circulation and migrate into tissues dependent on inflammatory triggers (51, 52), thus potentially adapting the pool of tissue-resident ILCs to local requirements. In accordance with this, we found a significantly decreased frequency of circulating pb ILC2s (defined as Lin^neg^CD127^+^CD161^+^CRTH2^+^ lymphoid cells) in patients suffering from various chronic inflammatory and fibrotic diseases affecting different organ systems (cystic fibrosis - CF, inflammatory bowel diseases - IBD and rheumatoid arthritis - RA) compared to healthy controls (Figures 1A,B). The applied flow cytometric strategy for the identification of human pb ILC2s was successfully validated by confirming the absence of T cells (TCR^+^, CD4^+^) and DCs (CD123^+^) in the indicated target cell population, which in addition showed positive staining for the common immune cell marker CD45 and the ILC2-associated transcription factor GATA3 (Figure S1A). Since the reduction of ILC2s in the blood of CF, IBD and RA patients was accompanied by a parallel decrease of total circulating ILCs (Figure 1C), it is unlikely that the observed effect can fully be explained by transdifferentiation of pb ILC2s into other ILC subsets, as described for ILC2s in nasal polyps of CF patients or COPD-affected lungs (30, 53). To explore the possibility that reduced systemic ILC2 frequencies in patients diagnosed for chronic inflammatory diseases (including CF, RA and IBD) are due to augmented tissue homing instead, we next focused on the chemokine receptor profile of circulating ILC2s. Examining a panel of defined chemokine receptors with a well-established function in inflammation-driven tissue homing of T cells (54, 55), we observed, in accordance with literature (56), that a high percentage of human pb ILC2s derived from healthy donors expressed CCR4 and CCR6. In contrast, the fractions of CCR5^+^ and especially CCR9^+^ and CXCR3^+^ pb ILC2s were markedly smaller (Figures S1B,C). Interestingly, we noted a significantly reduced frequency of CCR6^+^ cells within the circulating ILC2 fraction of CF, IBD and RA patients compared to healthy controls (Figure 1D), potentially implicating a general relevance of CCR6 for ILC2 homing into chronically inflamed tissue sites. In contrast to CCR6, neither the fraction of CCR4^+^, CCR5^+^, CCR9^+^, nor CXCR3^+^ pb ILC2s showed an altered frequency in the considered disorders (Figures 1E–H). And even though sex-specific differences in the frequency of ILC2s were suggested in asthmatic patients (57), the here observed phenomenon turned out to be gender-independent (Figure S1D).

**Figure 2:**
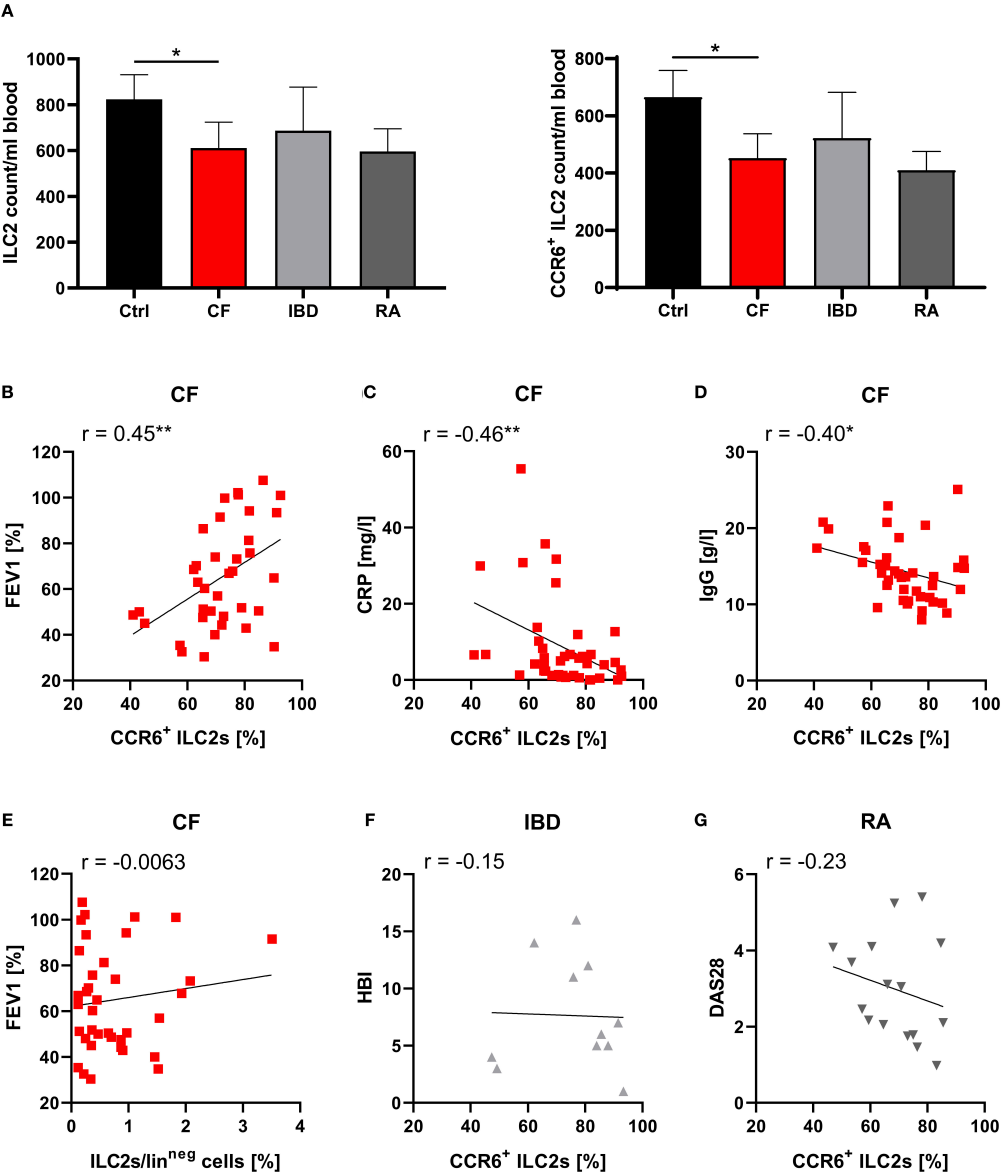
Specific role of CCR6^+^ pb ILC2s on disease severity in CF patients. **(A)** Total counts of human pb ILC2s and CCR6^+^ ILC2s per ml blood donated by healthy controls (ctrl, *n* = 40–41) and patients with CF (*n* = 35), IBD (*n* = 12–13), or RA (*n* = 17). Total counts were calculated based on PBMC numbers/ml blood (determined by Neubauer counting chamber) and the proportion of ILC2s and CCR6^+^ ILC2s among total PBMCs (determined by flow cytometry). The Mann–Whitney test was applied. **(B–D)** Correlation of the percentage of CCR6^+^ pb ILC2s and disease severity in CF patients based on FEV1% (B; *n* = 37), CRP (C; *n* = 41) and IgG serum levels (D; *n* = 41). The included raw data on CCR6 expressing pb ILC2s were partly also used in Figure 1D. **(E)** Correlation of the pb ILC2 frequency and FEV1% in CF patients (*n* = 37). **(F,G)** Correlation of the frequency of CCR6^+^ pb ILC2s and disease severity in Crohn's disease patients (F; Harvey-Bradshaw Index - HBI; *n* = 11) and RA patients (G; Disease Activity Score 28 - DAS28; *n* = 16). Spearman's *r* and regression lines are indicated. *p < 0.05, **p < 0.01.

On the level of absolute cell counts per defined blood volume, the observed ILC2 reduction as well as the decrease in CCR6^+^ ILC2s was clearly detectable in CF patients (Figure 2A). Notably, we found that phases of impaired respiratory function (determined by low levels of the forced expiratory volume in 1 s - FEV1%) and high inflammatory activity (determined by high C-reactive protein - CRP, and IgG levels) were associated with a more pronounced decrease of CCR6^+^ ILC2 frequencies (Figures 2B–D), while this phenomenon was not influenced by the type of the disease-underlying *CFTR* mutation (Figure S2A). The inverse correlation with disease severity was only seen in the subgroup of CCR6^+^ but not in total ILC2s (Figure 2E). Moreover, in contrast to CF patients, neither clinical exacerbation of IBD nor RA was associated with decreasing rates of circulating CCR6^+^ ILC2s (Figures 2F,G), indicating a distinct functional role of CCR6^+^ pb ILC2s in CF. And despite CF being often described as a Th17-dominated disorder (58), the disease-dependent reduction of CCR6^+^ pb ILCs was restricted to the ILC2 subset rather than ILC3s or ILC1s (Figures S2B,C). Taken together, the here depicted data collectively pointed to a potential role of CCR6 signaling during inflammation-triggered lung migration of circulating human ILC2s in CF.

### The CCR6 Ligand CCL20 Promotes Pulmonary Accumulation of Human pb ILC2s

**Figure 3:**
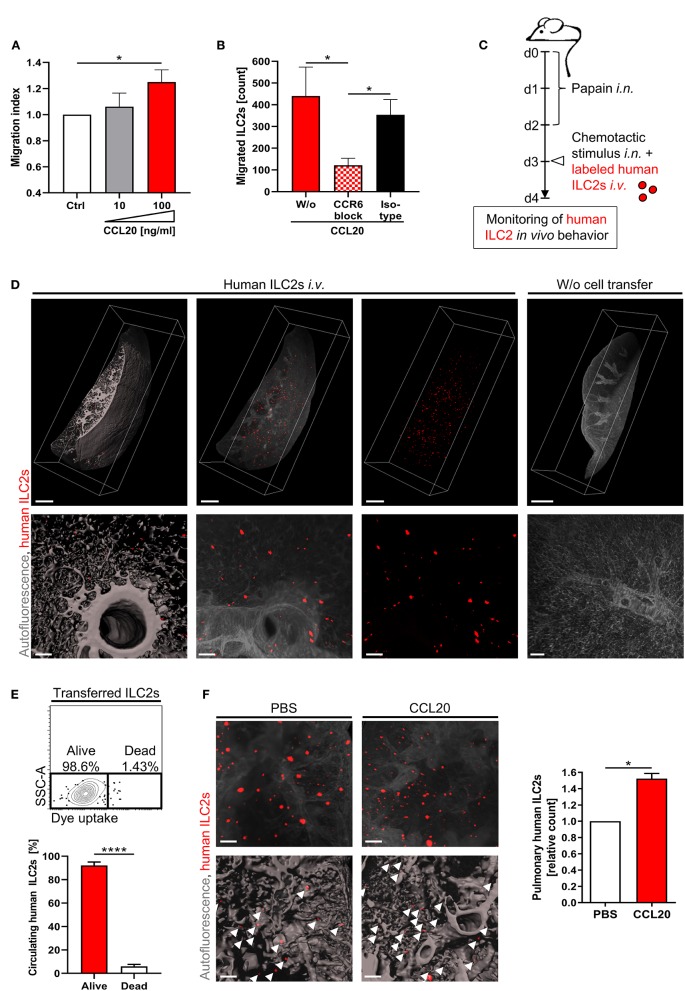
CCR6-CCL20-dependent accumulation of human pb ILC2s in the inflamed lung. **(A)** *In vitro* migration of CRTH2^+^ human PBMCs derived from healthy subjects towards CCL20. Migrated ILC2s (Lin^neg^CD161^+^CRTH2^+^ lymphoid cells) were flow cytometrically quantified in the bottom chamber (*n* = 9). **(B)** Effect of a CCR6 blocking antibody (50 μg/ml) on the *in vitro* migration of human ILC2s from control subjects toward CCL20 (100 ng/ml) (*n* = 9, isotype *n* = 3). **(C)** Experimental workflow of the *in vivo* lung homing assay. **(D)** Representative 3D-reconstructed LSFM images (≥ 11 independent experiments) of lung-accumulated human ILC2s (displayed in red). Overview (scale bars: 1,000 μm) and detailed images (scale bars: 100 μm) are depicted (left panel: surface mode, middle/right panels: maximum intensity projection - MIP). Mice without cell transfer served as control. **(E)** Viability of labeled human ILC2s in the murine blood 24 h after transfer; representative FACS plot and corresponding quantification (pooled data from *n* = 4 ctrl ILC2s and *n* = 3 CF ILC2s). The paired *t*-test was applied. **(F)** Pulmonary enrichment of *i.v*. injected labeled ctrl ILC2s after intranasal administration of CCL20 or PBS. Representative LSFM images (scale bars: 200 μm; top panel: MIP; bottom panel: surface mode) and corresponding quantification of accumulated labeled cells (indicated by arrows) (*n* = 3). Single outliers were detected by the Grubbs' test. The Mann-Whitney test was applied, if not indicated otherwise. For relative data the one-sample *t*-test was used. *p < 0.05, ****p < 0.0001.

While CCR6 represents a well-known mediator of T cell recruitment (59), its potential involvement in the directed movement of circulating ILC2s remained to be investigated. This aspect might be of particular relevance in CF, as affected patients showed increased levels of the CCR6 ligand CCL20 in BAL fluid (15). To study the role of CCL20 for ILC2 migration, we performed chemotaxis assays with primary human CRTH2^+^ pb cells. The CRTH2 ligand prostaglandin D2 (PGD2) and the CCR9 ligand CCL25 served as positive and negative control, respectively (Figures S3A,B), (31, 60). Performed analyses revealed a CCL20-induced enrichment of migrated Lin^neg^CD161^+^CRTH2^+^ ILC2s in the bottom compartment of the transwell (Figure 3A). Of note, the CCL20 concentration (100 ng/ml), which was found to successfully chemoattract human ILC2s in our experimental system (Figure 3A), was comparable to increased CCL20 protein levels reported in the airway surface liquid of CF patients (15). As the ILC2 migration in the presence of CCL20 was efficiently inhibited by antibody-mediated blockade of CCR6 (Figure 3B), our data identified CCR6 as crucial mediator of human ILC2 chemotaxis.

To gain further insights into the lung homing capacity of human pb ILC2s under *in vivo* conditions, we took advantage of a new humanized mouse model in which fluorescence labeled human ILC2s were intravenously transferred into recipient wildtype mice (49). Using light-sheet fluorescence microscopy (LSFM) imaging, this model allows to visualize the pulmonary accumulation of human ILC2s in the context of papain-induced airway inflammation (Figures 3C,D; Movies S1A,B). 24 hours after cell transfer, LSFM images of the murine lung depicted a pulmonary enrichment of human ILC2s, while transferred cells did not accumulate in the intestinal mucosa of recipient animals (Figure 3D; Figure S3C). Flow cytometric detection of transferred ILC2s in the blood of recipient mice ensured good survival of human ILC2s within the murine organism for the entire period of the experimental procedure (Figure 3E). In accordance with our *in vitro* data, intranasal administration of rh CCL20 prior to the transfer of fully labeled human ILC2s resulted in a significantly increased pulmonary accumulation of human ILC2s (Figure 3F; Figure S3D), while comparable numbers of human ILC2s in the blood of both groups confirmed equal numbers of initially transferred cells (Figure S3E). Overall, these data strongly suggest CCR6 signaling as potent chemotactic trigger for the directed migration of circulating human ILC2s toward inflammatory tissue sites in the lung.

### Pb-Derived Human ILC2s Drive Pulmonary Type-VI Collagen Production

**Figure 4:**
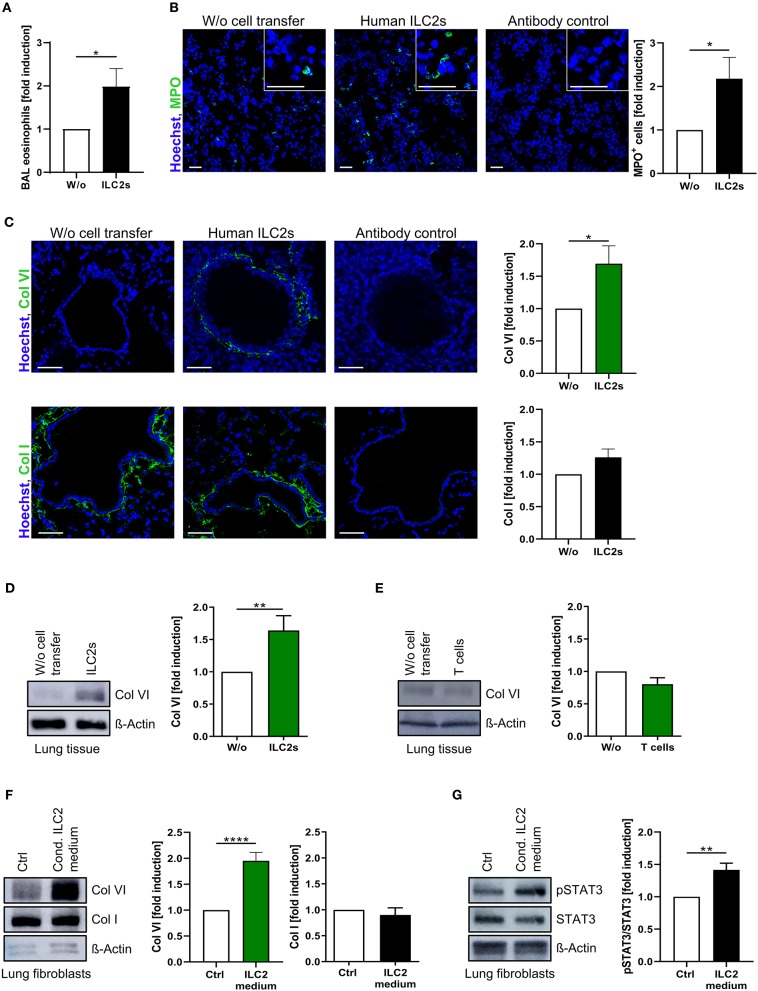
Induction of pulmonary Col VI expression by pb-derived ILC2s *in vivo* and *in vitro*. **(A)** Relative induction of murine BAL eosinophils (flow cytometrically determined as CD11c^neg^ SiglecF^+^ granulocytes) after *i.v*. injection of expanded human ILC2s in papain-treated mice (ctrl and CF ILC2s pooled, *n* = 13–21). **(B)** Representative images of murine lung tissue stained for MPO 24 h after intravenous ILC2 transfer (scale bars: 50 μm) and corresponding quantification of MPO^+^ cells (ctrl and CF ILC2s pooled, *n* = 11–15). **(C)** Representative images of murine lung tissue stained for Col VI (*n* = 14–21) and Col I (*n* = 18–24) 24 h after intravenous transfer of ctrl or CF ILC2s (scale bars: 50 μm) and corresponding quantifications of the peribronchial collagen/Hoechst signal intensities. **(D,E)** Western blot analysis of Col VI expression in lung tissue 24 h after *i.v* transfer of human ILC2s (D, ctrl and CF ILC2s pooled, *n* = 19–30) or CD4^+^ T cells (E, ctrl CD4^+^ T cells, *n* = 4–7); representative blots and corresponding quantifications. **(F,G)** Western blot analysis of Col VI (*n* = 20–26), Col I (*n* = 8–12) (F) and pSTAT3 (G; *n* = 8–11) expression in human lung fibroblasts treated for 48 h with 1:5 diluted ILC2-conditioned supernatants derived from *ex vivo* expanding human ctrl ILC2s; representative blots and corresponding quantifications. Fibroblasts stimulated with cytokines used for ILC2 expansion (IL-2, IL-33, IL-25, PHA) served as control. The one-sample *t*-test was applied. *p < 0.05, **p < 0.01, ****p < 0.0001.

The capacity of pb human ILC2s for pulmonary migration together with the observed association between decreased frequencies of circulating ILC2s and respiratory dysfunction in CF patients strongly implicated a direct impact of these cells on local fibro-inflammatory lung pathology. Indeed, ILC2-transferred recipient mice showed an increased frequency of eosinophils in the BAL and a significant pulmonary accumulation of neutrophils (Figures 4A,B). As the induction of airway eosinophilia represents a well-described consequence of lung ILC2 activation (61), these data indicated that transferred human ILC2s stably retained their type-2 immune function after pulmonary migration. Moreover, excessive neutrophilia as well as eosinophil activation represent hallmarks of human CF lung pathology (10, 62), suggesting an orchestrating function of blood-derived pulmonary ILC2s on local CF-associated lung inflammation. Besides the impact of ILC2s on inflammatory cell infiltrates, strikingly, we observed that the inflammation-driven accumulation of lung ILC2s relevantly influenced the composition of the extracellular matrix as well. *Ex vivo* analyses revealed a significantly upregulated protein expression of non-fibrillary type-VI collagen (Col VI) in lungs of ILC2-transferred recipient mice (Figures 4C,D), which could not be observed in extrapulmonary tissue (Figure S4A) or when human CD4^+^ T cells instead of ILC2s were administered intravenously (Figure 4E). This finding was of particular interest, as Col VI critically affects the pulmonary elasticity and its increased expression has been suggested to represent an early event within the pathogenesis of lung fibrosis (63, 64). In contrast, expression levels of fibril-forming type-I collagen (Col I), which represents the most abundant collagen type in lung tissue (65), was not found to be significantly upregulated in the presence of transferred human ILC2s (Figure 4C). In accordance with this *in vivo* observation, analyses of cultured primary human lung fibroblasts indicated a significant induction of Col VI expression after exposure to ILC2-conditioned cell culture medium, suggesting ILC2-secreted soluble factors as key mediators of this phenomenon (Figure 4F; Figure S4B). Furthermore, as an indicator of an advanced pro-fibrotic activation status (66), a marked upregulation of STAT3 phosphorylation was observed (Figure 4G), while the expression of Col I remained unaffected (Figure 4F). Overall, these findings support the concept that lung-entering ILC2s do not only orchestrate type-2 immune responses, but also secrete soluble mediators that activate Col VI production by pulmonary fibroblasts and induce subsequent tissue remodeling.

**Figure 5:**
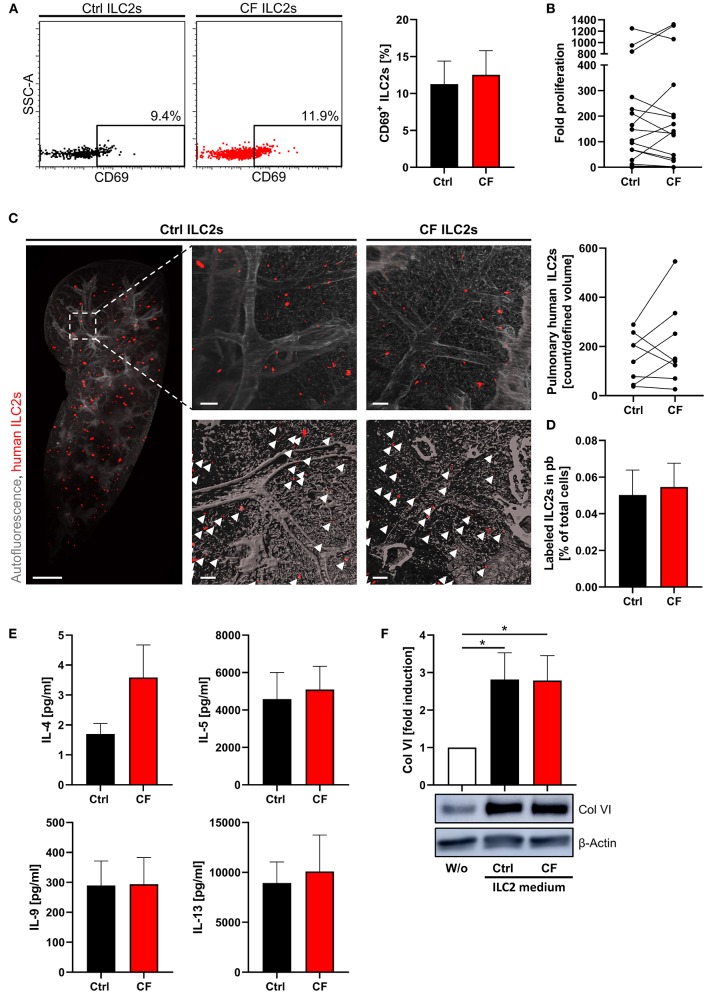
Unaltered potent *ex vivo* expansion, cytokine production and lung homing capacity of human CF ILC2s. **(A)** Percentage of CD69^+^ pb ILC2s in ctrl and CF subjects (*n* = 8–9) determined by flow cytometry; representative dot plots and corresponding quantification. **(B)** Fold proliferation of human pb-derived CF and ctrl ILC2s after 13–15 days of stimulation with PHA, IL-2, IL-33, and IL-25 in the presence of feeder cells (*n* = 17). **(C)** Representative LSFM images (3D reconstruction) of murine lung tissue with papain-induced inflammation 24 h after transfer of expanded labeled human CF or ctrl ILC2s and corresponding quantification of lung accumulated labeled human ILC2s (*n* = 8). Scale bars represent 1,000 μm for the overview image (MIP) and 100 μm for detailed images (top: MIP, bottom: surface mode). Labeled human ILC2s are displayed in red (indicated by arrows). **(D)** Corresponding quantification of labeled ILC2s in murine blood 24 h after *i.v*. transfer (*n* = 7). **(E)** Secreted cytokine levels by CF and ctrl ILC2s upon *ex vivo* stimulation (*n* = 8–16). Expanded and rested human ILC2s (adjusted cell numbers) were restimulated for 3 days with IL-2, IL-33, and IL-25; afterwards supernatants were collected for quantification of indicated cytokines via ELISA. Single outliers were detected by the Grubbs' test. **(F)** Western blot analysis of Col VI expression in human lung fibroblasts treated for 48 h with 1:5 diluted ILC2-conditioned supernatant derived from ctrl subjects and CF patients. Supernatants were harvested after 3 days of restimulating adjusted cell numbers of expanded human ILC2s; representative blot and corresponding quantification (*n* = 5–7). Fibroblasts stimulated with cytokines used for ILC2 restimulation (IL-2, IL-33, IL-25) served as control. The one sample *t*-test was applied. *p < 0.05.

Since the immune response underlying CF pathology has been suggested to be intrinsically affected by the inherited *CFTR* mutation (20), growing attention has been payed to the role of immune cells in CF. Therefore, we examined the function of circulating blood ILC2s in CF patients compared to healthy controls. In general, CF ILC2s showed a normal activation status and an unaltered capacity for expansion and lung migration after *ex vivo* stimulation (Figures 5A–D). Furthermore, all analyzed type-2 cytokines could clearly be detected in comparable levels in supernatants of restimulated CF and control ILC2s (Figure 5E), indicating an unchanged, but potent capacity of CF ILC2s for inflammatory cytokine production. In line with this, ILC2 conditioned supernatants derived from CF patients strongly induced Col VI expression in human lung fibroblasts to a similar extent as supernatants derived from simultaneously stimulated control ILC2s (Figure 5F). Thus, the inherited *CFTR* mutation in CF patients did not seem to influence ILC2 activity, leaving them functionally competent and with unaltered effector actions compared to ILC2s derived from healthy subjects.

### Additive Effects of ILC2-Derived IL-4 and IL-13 Induce Col VI Production in Fibroblasts

**Figure 6:**
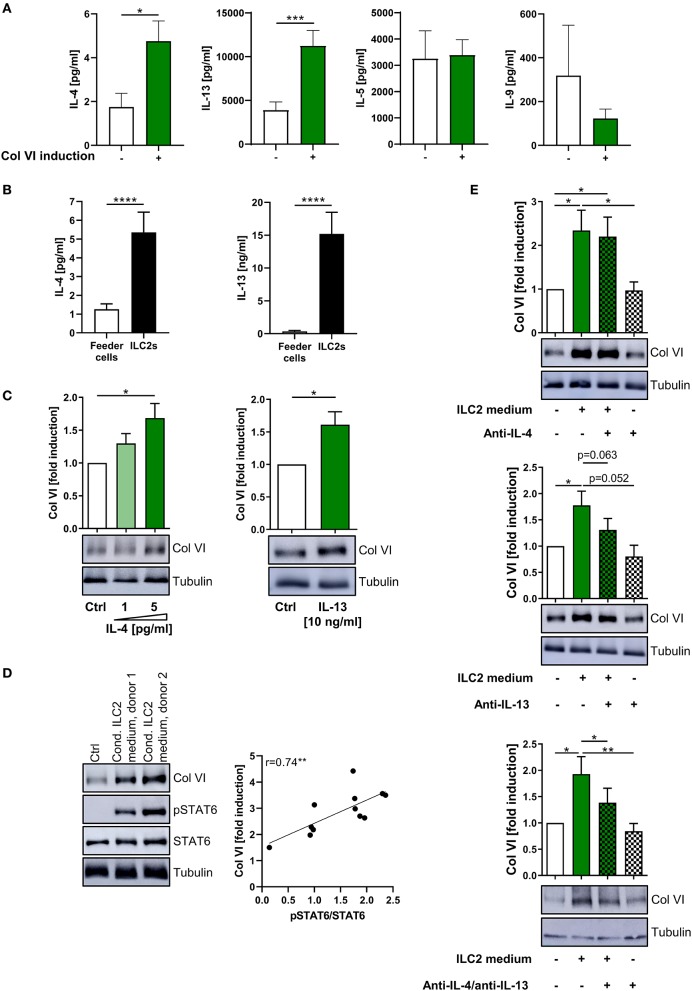
Additive effect of ILC2-derived IL-4 and IL-13 on pulmonary Col VI induction. **(A)** ILC2 conditioned supernatants were grouped according to their potency to induce Col VI expression in human lung fibroblasts (based on Western blot analyses) and were analyzed for concentrations of IL-4 (*n* = 5–32), IL-13 (*n* = 6–30), IL-5 (*n* = 6–31), and IL-9 (*n* = 4–21) via ELISA. The included raw data were partly also used in Figures 4F, 5E. Single outliers were detected by the Grubbs' test. The unpaired Student's *t*-test with Welch correction was performed. **(B)** IL-4 (*n* = 12–33) and IL-13 (*n* = 7–25) levels secreted by *ex vivo* expanding human ILC2s as determined by ELISA. The Mann-Whitney test was applied. **(C)** Representative Western blot analysis of Col VI expression in human lung fibroblasts stimulated with rh IL-4 (*n* = 5) and rh IL-13 (*n* = 10) and corresponding quantifications. The one-sample *t*-test was performed. **(D)** Western blot analysis of Col VI and pSTAT6 expression in human lung fibroblasts stimulated for 48 h with 1:5 diluted conditioned ILC2 supernatants collected from *ex vivo* expanding human ILC2s (d11–d13) (representative for *n* = 12). Fibroblasts stimulated with IL-2, IL-33, IL-25, and PHA in 1:5 diluted Yssel's medium served as control. Corresponding correlation of quantified pSTAT6/STAT6 signals and fold Col VI induction. The Pearson correlation coefficient was calculated. **(E)** Representative Western blot analysis of Col VI expression in human lung fibroblasts stimulated with conditioned ILC2 supernatants as well as anti-IL-4 (10 ng/ml) and anti-IL-13 (20 ng/ml) as indicated (*n* = 6–9) and corresponding quantifications. The paired two-tailed Student's *t*-test was applied. *p < 0.05, **p < 0.01, ***p < 0.001, ****p < 0.0001.

As the observed Col VI-inducing capacity of ILC2s appeared to be driven by their secretome (Figures 4F, 5F), we compared ILC2-conditioned supernatants derived from different ILC2 donors with regard to the concentrations of specific type-2 cytokines and their Col VI-inducing capacity. The performed studies indicated that ILC2-conditioned medium, which potently induced Col VI expression in lung fibroblasts, contained significantly increased levels of IL-4 and IL-13 compared to medium, which failed to induce Col VI expression (Figure 6A). In contrast, IL-5 and IL-9 concentrations did not show a similar association with induced levels of Col VI (Figure 6A). In accordance with these findings, exposure of lung fibroblasts to recombinant IL-4 or IL-13, in concentrations comparable to those detected in the supernatants of stimulated ILC2s, resulted in a significant induction of Col VI expression (Figures 6B,C). Moreover, conditioned ILC2 media substantially induced STAT6 phosphorylation in fibroblasts (Figure 6D), which is known to be exclusively activated in response to IL-4Rα signaling (67). Since both, the IL-4 and IL-13 receptor share the IL-4Rα subunit, this finding further indicated that ILC2-mediated Col VI induction is conveyed via IL-4 and IL-13 receptor signaling. Indeed, the extent of STAT6 activation significantly correlated with the intensity of Col VI induction (Figure 6D). Finally, the functional involvement of IL-4 and IL-13 in the postulated ILC2 – fibroblast – Col VI axis could be confirmed by the observation that combined antibody-mediated blockade of both cytokines was able to significantly dampen the Col VI-inducing capacity of the ILC2 secretome on pulmonary fibroblasts (Figure 6E). In the same experimental setting, selective blockade of either IL-4 or IL-13 alone only resulted in mild and non-significant effects on Col VI expression (Figure 6E). Hence, these analyses strongly suggested that additive effects of secreted cytokines, such as IL-4 and IL-13, on local fibroblast activation and Col VI production crucially mediate the tissue modulating function of pulmonary ILC2s.

## Discussion

Pulmonary fibrosis and persistent inflammation represent the key drivers of morbidity and mortality among CF patients. So far, the molecular mechanisms of CF lung injury are, however, only incompletely understood. In the present study, using a new humanized mouse model, we uncovered the capacity of circulating human ILC2s to follow a CCL20 gradient, accumulate in inflamed lung tissue and drive local pulmonary Col VI deposition. Moreover, ILC2-derived cytokines and in particular additive effects of IL-4 and IL-13 were identified as mediators of Col VI production by lung fibroblasts. Based on the correlation of reduced numbers of CCR6^+^ ILC2s in the blood circulation of CF patients with enhanced lung inflammation and the previously described presence of pathologically increased CCL20 levels in CF BAL (15), we postulate the CCR6 - CCL20-driven ILC2 lung homing mechanism to be important in driving CF lung pathology.

In addition to the preferential accumulation of ILC2s at mucosal surfaces, like in the lung, gut and skin (68), it is also well-established that ILC precursors and even mature ILC2s are present in the blood stream of healthy individuals and patients with inflammatory diseases (22, 69–71). However, the question about the role of these circulating ILCs is still insufficiently answered, even though local pulmonary inflammation is well-established to be reflected in altered ILC2 numbers in the peripheral blood circulation (72–74). So far, there have been only very few studies on a functional involvement of human pb ILC2s in systemic immune responses or their capacity to home to specific tissue sites in order to increase the local pool of resident ILC2s (75). Based on the here presented data, we postulate that in CF, CCR6^+^ ILC2s are chemotactically attracted from the peripheral blood into inflamed pulmonary tissue sites, where they influence local disease progression. Using parabiotic mouse models, murine ILC2s have initially been described to be mainly tissue-resident under acute inflammatory states, whereas their homing capacity has already been indicated under chronic inflammatory conditions (76). The latter seems to be consistent with the recently described infection- or inflammation-triggered inter-organ migration of gut resident ILC2s via S1P-mediated chemotaxis and the arising idea of a milieu-dependent local recruitment and tissue-specific ILC-poiesis (51, 69, 75). In addition to S1P-driven chemotaxis, the CRTH2 ligand PGD2, the CCR4 ligand CCL22 and IL-33 have been described as potent mediators of chemotactic ILC2 attraction (31, 60, 77, 78). Our *in vitro* and *in vivo* data now additionally identified the chemotactic potential of CCL20 on human ILC2s. As IL-33 and CCL20 are both upregulated in the lung of CF patients (15, 44, 79), these findings suggest a joint chemotactic ILC2 recruitment into the inflamed lung in states of exacerbated CF. Taking into account that CCL20 expression can also be upregulated in other organs under inflammatory conditions (80, 81) it cannot be completely excluded that CCR6^+^ ILC2s might partly also migrate to other CF-affected organs besides the lung. However, as the described 90-fold induction of CCL20 protein levels in the BAL of CF patients compared to healthy volunteers nearly doubled a 47-fold upregulation of CCL20 mRNA expression in the gut of patients suffering from ulcerative colitis, (15, 80) this indicates a particular relevance of the CCL20 - CCR6 axis for lung homing in CF. Moreover, a significant association between reduced blood CCR6^+^ ILC2 counts and increased disease activity was observed in CF patients but in none of the other analyzed fibro-inflammatory diseases affecting the gut or joints, further strengthening an organ- and CF-specific role of CCR6^+^ ILC2s. Besides the increased presence of chemotactic triggers in CF airways, the intrinsic lung homing capacity of CF ILC2s appeared to be independent of the inherited *CFTR* mutation. Based on the here identified chemotactic potential of CCL20 on human ILC2s, we suggest that decreased numbers of CCR6^+^ ILC2s in the peripheral blood of CF patients reflect the enhanced attraction of these cells to the site of inflammation. Of course, the experimental validation of this concept in humans was limited to some extend by the fact that we were unable to directly compare and correlate results from blood-derived circulating ILC2s with the number and activation profile of ILC2s in lung tissue of the same patients due to the lack of corresponding lung tissue or BAL samples. However, in the context of aspirin-exacerbated respiratory disease as well as *Mycobacterium tuberculosis*-infected lungs, published data indicated that the local accumulation of ILC2s in mucosal tissue was associated with a parallel reduction of blood ILC2 percentages (72, 82). A similar impact of tissue-migrating immune cells on the number of their circulating counterparts has also been postulated for T cells in the context of IBD (83). Moreover, the *in vivo* ability of human blood ILC2s to accumulate in the inflamed lung tissue upon CCL20-mediated chemoattraction was proven in a newly established humanized mouse model. In the light of described inter-species differences between murine and human ILC2s (70, 84, 85) this humanized model of lung inflammation represented a clear advantage over classic mouse models and allowed us to directly analyze patient-derived primary human ILC2s under inflammatory *in vivo* conditions. Of particular relevance for the here described findings, there exist, to our knowledge, no published data on a potential CCR6 expression of circulating murine ILC2s, while in tissue-infiltrating murine helper ILCs, CCR6 expression seemed to be mainly restricted to ILC3s (86). Together with the observation that human and murine ILC2s differ in the expression profile of the prostaglandin D2 receptor CRTH2 (84), this potentially implicates that the migratory behavior of ILC2s is differentially regulated between both species. Collectively, the results acquired in the humanized *in vivo* model together with our human association data pointed to a relevant CCL20-triggered lung recruitment of systemic human ILC2s under the distinct pathologic conditions of CF.

Generally, neutrophilia and an IL-17 signature have been described as common hallmarks of CF-underlying pathomechanisms (58), although several studied also pointed to a relevant, but less acknowledged, skewing of immune responses towards type-2 mediated effects (87). Of particular interest in this context, a recently published study postulated the preferential transdifferentiation of ILC2s into IL-17 secreting ILC3-like cells in nasal polyps of CF patients (53). However, our own *in vivo* data demonstrated the retention of important type-2 functions of pb-derived ILC2s in inflamed lung tissue, indicated by a potent induction of lung eosinophilia, although we cannot exclude that transdifferentiation into ILC3-like cells might potentially occur at a later time point. Regarding the involvement of tissue-infiltrating neutrophils in the pathogenesis of CF lung disease, our observation that intravenously transferred human ILC2s resulted in increased pulmonary neutrophil accumulation in the humanized mouse model, might suggest an active contribution of ILC2s to the CF-related neutrophil recruitment into chronically inflamed pulmonary tissues sites. Thus, future studies are needed to further elucidate the molecular mechanisms underlying the ILC2-mediated numeric regulation of pulmonary neutrophils and their potential interference with the classically described IL-17-driven chemotaxis of neutrophils in CF patients (88).

**Figure 7:**
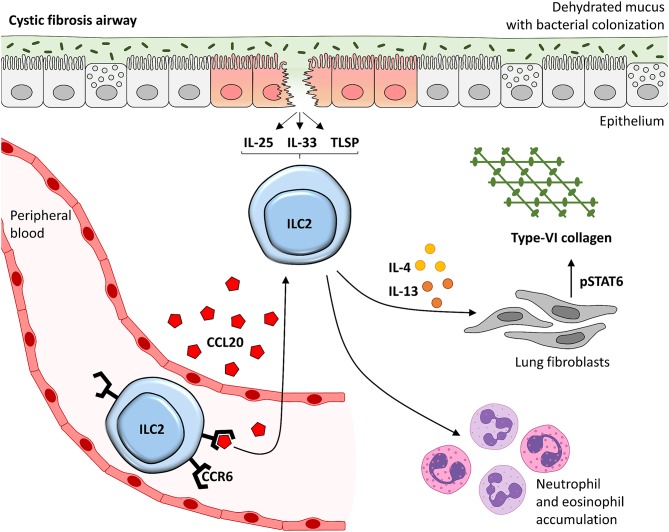
Suggested model of the CCL20 - ILC2 – IL-4/IL-13 – Col VI axis in lung disease of CF patients. CCR6 expressing ILC2s in the peripheral blood stream are chemoattracted into the inflamed lung tissue of CF patients in a CCL20-dependent manner. Locally accumulated and activated pb-derived ILC2s likely induce type-2 mediated lung inflammation, including neutrophils and eosinophil accumulation, but also pulmonary Col VI expression in lung fibroblasts. This is mediated via the secreted mediators IL-4 and IL-13 in particular which signal via STAT6. This might finally lead to local tissue remodeling potentially driving disease progression in CF patients. TSLP, thymic stromal lymphopoietin.

Again by using the novel humanized mouse model of ILC2 homing to the inflamed lung, we noted that the pulmonary accumulation of human ILC2s drives Col VI production *in vivo*. Indeed, pulmonary ILC2s are preferentially located in collagen-rich areas close to the airway epithelium (27, 29) immediately subjacent to Col VI (63, 89, 90) that functions as an important anchoring element linked to the basement membrane. Moreover, expression of Col VI is known to be increased during lung fibrosis (63), and subepithelial fibrosis and thickening of the reticular basement membrane have been described as important morphological alterations in CF lungs (91, 92). These findings support the pathogenic relevance of the described interplay between accumulated ILC2s, fibroblasts and Col VI for pulmonary tissue remodeling in chronic lung manifestations of CF. In line with the general concept that the pro-fibrotic function of activated ILC2s is driven by their capacity to release high amounts of type-2 cytokines (35, 42), it has recently been postulated that ILC2-derived IL-9 might support pulmonary inflammation in CF patients via activation of mast cells and subsequent release of TGF-β (14). However, we did not observe a significant association of increased IL-9 secretion and the capacity of human ILC2s to induce Col VI expression in human lung fibroblasts. Thus, our findings strongly suggest that other cytokines or mediators also contribute to the ILC2-mediated pathologic lung tissue remodeling in CF patients. Accordingly, we identified the two closely related cytokines IL-4 and IL-13, that both signal via downstream activation of STAT6 and STAT3 (67, 93), as key mediators of the ILC2-induced Col VI production. Notably, the here postulated functional relation between ILC2-triggered pulmonary tissue remodeling and the quality of CF lung disease is in accordance with the observation that increased type-2 cytokine levels in the BAL of CF patients significantly correlated with pathologic lung tissue alterations as assessed by high-resolution computed tomography (46). Overall, our data thus provide evidence for a crucial involvement of pb-derived human ILC2s in the pathogenesis of CF (Figure 7). In addition, the here described ILC2 – IL-4/IL-13 – Col VI axis elucidates a new aspect of their tissue modulating capacity, while our findings on CCL20-driven ILC2 chemotaxis highlight the relevance of the systemic ILC2 pool for the clinical course of CF. Although future studies are needed to confirm the strict CCR6-dependency of CCL20-triggered ILC2 lung homing under CF-relevant *in vivo* conditions, our results open new therapeutic avenues for the treatment of CF or other fibro-inflammatory lung diseases, potentially allowing a numeric regulation of local ILC2 pools in the inflamed lung tissue.

## Data Availability Statement

The datasets generated and analyzed during the current study are available from the corresponding author on reasonable request.

## Ethics Statement

The studies involving human participants were reviewed and approved by Local ethical committee and the institutional review board of the University of Erlangen-Nuremberg, Germany. The patients/participants provided their written informed consent to participate in this study. The animal study was reviewed and approved by Government of Lower Franconia, Germany.

## Author Contributions

AS-K, VG, MD, and LK performed the experiments. AS-K, KH, RA, SZi, FF, SZu, RL-P, CN, AR, AK, AG, ES, SW, MN, and IA provided clinical samples, protocols, reagents, or designed experiments. AS-K, MN, and IA analyzed and interpreted the data, and drafted the manuscript. All authors critically revised the manuscript for important intellectual content.

## Conflict of Interest

MN has served as an advisor for Pentax, Giuliani, MSD, Abbvie, Janssen, Takeda and Boehringer. The remaining authors declare that the research was conducted in the absence of any commercial or financial relationships that could be construed as a potential conflict of interest.
