# Supplementary material for: ILC2 Lung-Homing in Cystic Fibrosis Patients: Functional Involvement of CCR6 and Impact on Respiratory Failure
Source: Front Immunol. 2020 May 7;11:691. doi: 10.3389/fimmu.2020.00691 (PMC7221160; doi:10.3389/fimmu.2020.00691)
Supplement: Supplementary file 1 [file Data_Sheet_1.docx]

Supplementary Material





**Figure S1: Specific identification of human pb ILC2s as Lin^neg^CD127^+^CD161^+^CRTH2^+^ lymphoid cells.**

Flow cytometric characterization of human pb ILC2s (gated as Lin^neg^CD127^+^CD161^+^CRTH2^+^ lymphoid cells). Specifically stained ILC2s are shown in red and a combination of the respective fluorescence-minus-one (FMO) and isotype controls in blue. Numbers indicate frequencies of ILC2s expressing the analyzed factor. **(A)** Expression of CD4 (n = 18), TCRα/β and TCRγ/δ (n = 22), CD123 (n = 25) as well as CD45 (n = 5) and GATA3 (n = 6) on Lin^neg^CD127^+^CD161^+^CRTH2^+^ vs. Lin^+^ lymphoid cells or total PBMCs; representative gating and corresponding quantification. Statistical significance was calculated using the paired Student´s t-test. ∗∗∗∗p < 0.0001. **(B)** Representative gating of the CCR4, CCR5, CCR6, CCR9 and CXCR3 expression on ILC2s and **(C)** corresponding quantification in healthy volunteers (n = 30-49). **(D)** Gender-specific frequency of CCR6^+^ ILC2s in the peripheral blood of healthy ctrl subjects (n = 16-33) as well as CF (n = 13-27), IBD (n = 4-13) and RA patients (n = 6-11).





**Figure S2: ILC2-specific, mutation independent decrease of the CCR6^+^ cell fraction in the peripheral blood of CF patients.**

**(A)** Mutation-dependent frequency of CCR6^+^ blood ILC2s (identified as Lin^neg^CD127^+^CD161^+^CRTH2^+^ lymphoid cells) in CF patients with homozygous (F508del/F508del, n = 19) or heterozygous deletion of phenoylalanin at the amino acid position 508 (F508del/xx, n = 16) and those with less common mutations (xx/xx, n = 5). **(B)** Flow cytometric characterization of the CCR6 expression on blood ILC subgroups. Representative gating of ILC1s (Lin^neg^CD127^+^CD7^+^CD117^-^CRTH2^-^ lymphoid cells), ILC2s (Lin^neg^CD127^+^CD7^+^CRTH2^+^ lymphoid cells) as well as ILC3s (Lin^neg^CD127^+^CD7^+^CD117^+^CRTH2^-^ lymphoid cells) and their respective CCR6 expression is shown. Numbers indicate frequencies of the parent population. **(C)** Corresponding quantification of CCR6^+^ ILC3s, ILC1s and ILC2s in ctrl subjects (n = 11-12) and CF patients (n = 17-19). The unpaired Student´s t-test was applied. ∗p < 0.05.





**Figure S3: Lung-specific enrichment of human pb ILC2s.**

**(A, B)** Transmigration assay with freshly isolated pb CRTH2^+^ cells towards PGD2 (A, 10 nM, n = 12) and CCL25 (B, 100 ng/ml, n = 7) serving as positive and negative control, respectively. Lin^neg^CD161^+^CRTH2^+^ ILC2s in the bottom well were enumerated flow cytometrically. The one-sample t-test was applied. ∗p < 0.05. **(C)** Analysis of the *in vivo* accumulation of expanded labeled human pb ILC2s in the gut and inflamed lung tissue of papain-treated C57BL/6 mice 24 h after *i.v.* transfer. Representative 3D reconstruction of lung tissue and ileum showing *i.v.* injected human ILC2s in red; LSFM overview images (scale bars: 300 µm; lung: surface mode, ileum: blend mode) and detailed images (scale bars: 100 µm; MIP) are displayed (n = 4). **(D)** Representative FACS plots showing the label efficiency of ILC2s prior to injection and **(E)** the percentage of labeled ILC2s in the peripheral blood 24 h after *i.v*. injection in mice treated intranasally with control PBS or rh CCL20; representative dot plots and corresponding quantification (n = 3).





**Figure S4: Lung- and ILC2-specific Col VI induction.**

**(A)** Western Blot analysis of Col VI expression in lung tissue and ileum 24 h after intravenous ILC2 transfer into mice with papain-induced lung inflammation (n = 2, 1 experiment). **(B)** Western blot analysis of Col VI induction by soluble factors derived from ILC2 stimulation experiments. Primary human lung fibroblasts were treated with 1:5 diluted conditioned supernatants derived from *ex vivo* expanding human ILC2s in the presence of γ-irradiated feeder cells or stimulated feeder cells alone; unconditioned medium supplemented with ILC2 stimulation cytokines served as control (ctrl); representative blot and corresponding quantification (n = 8-9). The one-sample t-test was applied. ∗p < 0.05.

**Table S1: Overview of analyzed subjects.**

**
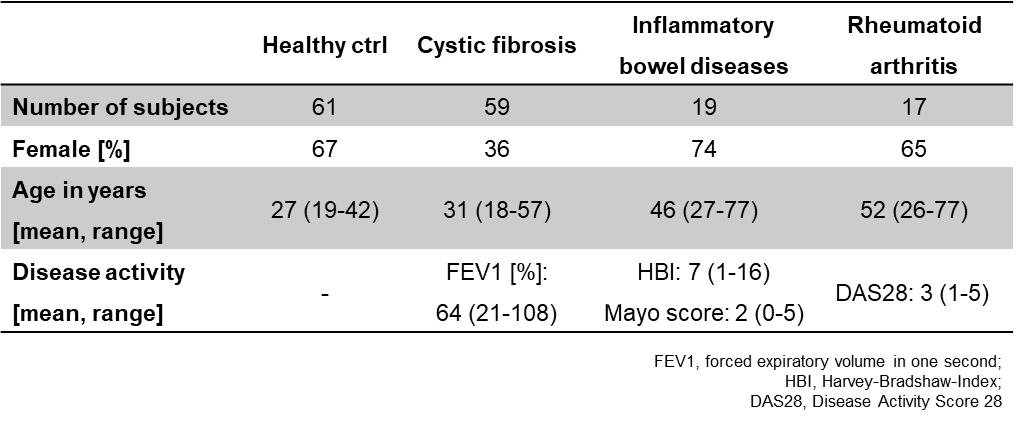
**

**Movie S1: Light-sheet fluorescence microscopy of lung-accumulated pb ILC2s.**

Representative animation of 3D reconstructed lung lobes, 24 h after *i.v.* transfer of labeled human ILC2s into mice with papain-induced lung inflammation. Imaging was performed by LSFM. **(A)** Overview of a lung lobe displayed as MIP. **(B)** Detailed image of a lung lobe displayed as MIP in the beginning and surface mode in the end. The here presented lung lobe is also shown in **Figure 5C**. Autofluorescent tissue architecture is displayed in grey, lung-accumulated human ILC2s in red**.**
